# Potential for trans-pulmonary tumor markers in the early diagnosis of lung cancer: a case report

**DOI:** 10.1186/s12890-024-03288-z

**Published:** 2024-09-18

**Authors:** Ken Monahan, Michael Kammer, Yan Ru Su, Wade Iams, Eric Grogan, Fabien Maldonado

**Affiliations:** 1 Division of Cardiovascular Medicine, Vanderbilt University Medical Center, 1215 21st Avenue – Medical Center East – 5th Floor, Nashville, TN 37232 USA; 2 Division of Allergy, Pulmonary, and Critical Care Medicine, Vanderbilt University Medical Center, Nashville, TN USA; 3 Division of Hematology and Oncology, Vanderbilt University Medical Center, Nashville, TN USA; 4 Department of Thoracic Surgery, Vanderbilt University Medical Center, Nashville, TN USA

**Keywords:** Tumor markers, Trans-pulmonary samples, Lung cancer

## Abstract

**Background:**

Measurement of tumor markers from peripheral venous blood is an emerging tool to assist in the early diagnosis of lung cancer. Samples from the pulmonary artery and pulmonary artery wedge position (trans-pulmonary samples) are accessible via right-heart catheterization and, by virtue of their proximity to lung tumors, may increase diagnostic yield.

**Case presentation:**

We report a case of a 64 year-old woman from whom trans-pulmonary samples were obtained and who was diagnosed 16 months later with recurrent metastatic small cell lung cancer. Carcinoembryonic antigen, cytokeratin fragment 21 − 1 (CYFRA), and human epididymis protein 4 (HE4) levels demonstrated increasing concentrations across the pulmonary circulation. These gradients exceeded the assays’ coefficient of variation by several-fold. For CYFRA and HE4, pulmonary artery wedge concentrations exceeded peripheral venous levels by more than 10% and peripheral arterial levels were up to 8% higher than peripheral venous levels.

**Conclusions:**

Evaluating the feasibility and utility of trans-pulmonary tumor markers for lung cancer diagnosis in a larger cohort should be considered. The addition of a peripheral arterial sample to standard peripheral venous samples may be a more practical alternative.

## Background

Early detection of lung cancer using circulating tumor biomarkers is an increasingly robust area of investigation [1] and has been demonstrated prior to clinical diagnosis [2]. Blood for analysis is typically obtained from a peripheral vein, though trans-organ samples have been used to characterize tumor metabolism in non-pulmonary sites [3]. Trans-pulmonary samples, taken from the pulmonary artery (PA) and pulmonary artery wedge (PAW) position, are accessible via standard right-heart catheterization, but their use for early detection of lung cancer has not been investigated. Markers of lung cancer may be more concentrated in the pulmonary circulation, due to proximity to the tumor site, and thus could enhance detection relative to contemporaneous peripheral venous samples. We report a case where peripheral and trans-pulmonary samples were obtained from a patient who was subsequently diagnosed with recurrent metastatic small cell lung cancer (SCLC) more than a year after sample acquisition.

## Case presentation

A 64 year-old woman presented to the cardiac catheterization laboratory for elective right and left-heart catheterization to evaluate persistent shortness of breath. Six years prior, she was treated with radiation, carboplatin, and etoposide for SCLC localized to the left lower lobe and had been in complete clinical remission. She provided informed consent to participate in a study of trans-pulmonary biomarkers in pulmonary hypertension [4, 5].

Peripheral samples were obtained from the venous and arterial sheaths used for catheterization access (right internal jugular vein and right radial artery, respectively). For trans-pulmonary sampling, a 7 French Swan-Ganz catheter was placed in the PA or PAW position. Catheter location was confirmed by pressure waveform analysis. The samples were collected in BD Vacutainer tubes with K2 EDTA and immediately placed on ice, then hand-delivered to our Cardiology Core Laboratory for Translational and Clinical Research. Plasma was separated by centrifugation at 1500 g for 10 min and stored in a -80 °C freezer until use for the tumor marker testing.

Multiple chest x-rays and computed tomography (CT) scans over the next year did not reveal any findings concerning for malignancy. Sixteen months after trans-pulmonary samples were obtained, a chest CT revealed multiple lung nodules and a moderate left-sided pleural effusion. A full-body positron emission tomography scan showed a hypermetabolic left hilar mass and hypermetabolic pleural, hepatic, and adrenal lesions. Ultrasound-guided biopsy of a liver nodule showed small cell carcinoma, presumably metastatic from the lung mass.

**Fig. 1:**
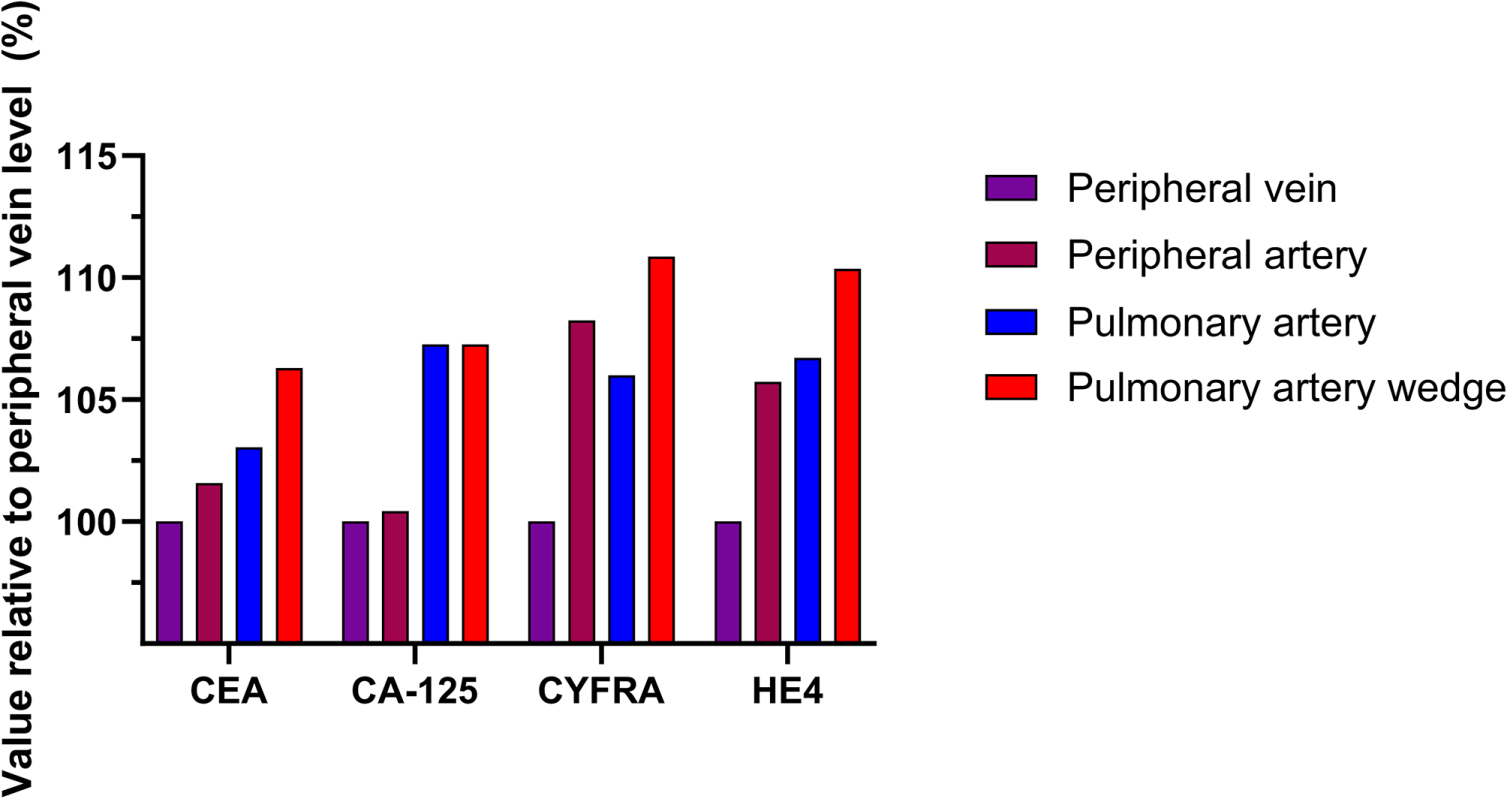
Tumor marker values relative to peripheral venous levels For CEA, CYFRA, and HE4, the PA-PAW gradient exceeds the marker’s coefficient of variation by several-fold. The peripheral vein to peripheral artery gradient is comparable to the trans-pulmonary gradient for CYFRA and HE4 CEA: carcinoembryonic antigen CYFRA: cytokeratin fragment 21 − 1 HE4: human epididymis protein 4 PA: pulmonary artery PAW: pulmonary artery wedge

Nearly 10 years after the samples were collected, carcinoembryonic antigen (CEA), cancer antigen 125 (CA-125), cytokeratin fragment 21 − 1 (CYFRA), and human epididymis protein 4 (HE4) levels were measured [6] using a Roche Cobas e411 analyzer. On reproducibility testing performed with this machine prior to analyzing the case samples, the coefficients of variation (CV) for the tumor markers were as follows: CEA: 1.3–1.8%, CA-125: 1.0-1.4%, CYFRA: 1.6–2.5%, HE4: 1.0-1.1%. The absolute values of each marker at each site are shown in Table 1 and the values relative to the peripheral venous levels of each marker are shown in Fig. 1. The venous-arterial gradient (relative to the peripheral venous level) exceeded the CV for CYFRA (by ∼ 3-fold) and HE4 (by nearly 6-fold) and the PA-PAW gradient (relative to the PA level) exceeded the CV for CEA (by ∼ 2-fold), CYFRA (by ∼ 2-fold), and HE4 (by ∼ 3-fold). For CEA, CYFRA, and HE4, values at all sites were above the cutoff that typically denotes a positive test whereas for CA-125, no value at any site exceeded the threshold for a positive result [6].

**Table 1:** Tumor markers at peripheral and trans-pulmonary sites

Site	CEA (ng/mL)	CA-125 (U/mL)	CYFRA (ng/mL)	HE4 (pM/L)
PAW	10.13	20.53	2.96	169.4
PA	9.82	20.53	2.83	163.8
Peripheral artery	9.68	19.22	2.89	162.3
Peripheral vein	9.53	19.14	2.67	153.5

CA-125: cancer antigen 125

CEA: carcinoembryonic antigen

CYFRA: cytokeratin fragment 21 − 1

HE4: human epididymis protein 4

PA: pulmonary artery

PAW: pulmonary artery wedge

Commonly used cut-off values for these markers (i.e. value above which the test is considered ‘positive’ for malignancy) are: CEA: 5 ng/mL; CA-125: 35 U/mL; CYFRA: 2.4 ng/mL; HE4: 97 pM/L [6]

## Discussion and conclusions

This case raises the possibility that trans-pulmonary tumor marker analysis may be useful in the early diagnosis of lung cancer and, by extrapolation, perhaps in the evaluation of response to treatment as well. In 3 of the 4 markers tested, the trans-pulmonary gradient exceeded by several fold the coefficient of variation, suggesting that the gradient was less likely due to inherent variability in measurement. The trans-pulmonary gradients for these markers ranged from ∼ 3–5%, which may be a large enough difference to cross the threshold between a ‘negative’ and ‘positive’ result, though that phenomenon was not demonstrated in this case. Even for the marker with no appreciable trans-pulmonary gradient (CA-125), the PA and PAW levels were considerably higher than their peripheral counterparts suggesting that sampling closer to the source of the tumor may have diagnostic utility. The peripheral vein to peripheral artery gradient exceeded the trans-pulmonary gradient for two of the markers, suggesting that adding a peripheral arterial sample to routine venipuncture may be a reasonable, and more practical, surrogate for trans-pulmonary samples. The direction of the gradient suggests increased shedding of tumor marker across the pulmonary circulation, which is physiologically plausible.

Variation in trans-pulmonary gradients between markers, as shown here, could be leveraged to create enhanced individualized tumor marker signatures or profiles and thus provide additional insight into the fundamental mechanisms of tumor biology and the lung’s response to it, which may help guide management longitudinally.

There are, at present, no validated lung cancer-specific markers available in routine clinical care. However, each of the markers analyzed for this report has been found to be elevated in lung cancer relative to both healthy controls and in those with benign pulmonary nodules [1, 7–9]. As such, we posited that these markers would be reasonable to include in this initial ‘proof-of-concept’ test of our hypothesis that concentrations of lung cancer-associated antigens vary across the pulmonary circulation.

This case introduces the potential added value of trans-pulmonary sampling compared to standard peripheral venous tumor marker analysis for the early detection of lung cancer. Future work should focus on assessing the feasibility and safety of adding a peripheral arterial site in a larger cohort, evaluating additional markers, and potentially expanding the trans-organ sampling concept to other organs/malignancies.

## Data Availability

Data are provided within the manuscript and are available from the corresponding author on reasonable request.

## Abbreviations

CA-125: Cancer antigen 125
CEA: Carcinoembryonic antigen
CT: Computed tomography
CV: Coefficient of variation
CYFRA: Cytokeratin fragment 21 − 1
HE4: Human epididymis protein 4
PA: Pulmonary artery
PAW: Pulmonary artery wedge
SCLC: Small cell lung cancer
